# The potential hazard of drug-eluting stent-induced coronary vasospasm causing subacute stent thrombosis: a case report

**DOI:** 10.1186/s12872-016-0410-4

**Published:** 2016-11-25

**Authors:** Hiroki Shibutani, Yuzo Akita, Yumie Matsui, Masahiro Yoshinaga, Masahiro Karakawa

**Affiliations:** Division of Cardiology, Osaka Saiseikai Izuo Hospital, 3-4-5 Kitayama, Taisho-ku, Osaka 551-0032 Japan

**Keywords:** Percutaneous coronary intervention, Effort angina, Vasospastic angina, Stent-edge spasm, In-stent thrombosis

## Abstract

**Background:**

Drug-eluting stent (DES) -induced coronary vasospasm is a well known phenomenon after stent implantation; however, the extent of this risk is still unknown. We report a case in which DES-induced severe coronary vasospasm was clinically suspected as a cause of subacute stent thrombosis (ST).

**Case presentation:**

A 67-year-old man came to our hospital due to chest pain with mild exercise. He was diagnosed with effort angina by coronary angiography and underwent DES implantation in the mid-left ascending artery (LAD) after the administration of dual anti-platelet therapy. The procedure was uneventful, but his symptoms changed from effort angina to rest angina after stenting. Five days after the procedure, subacute ST occurred, requiring aspiration thrombectomy and balloon angioplasty. Thereafter, he continued to report early morning chest discomfort. We performed a spasm provocation test to evaluate the coronary vasomotor response; it revealed severe stent-edge spasm in the left main trunk to the LAD, except for the stented lesion, and total occlusion of the left circumflex artery.

**Conclusions:**

To our knowledge, the present case is the first report describing in-stent thrombosis secondary to stent-edge spasm. This case describes the potential hazard of DES-induced coronary vasospasm. Although there are several overlapping risk factors for ST development, we consider that stent-edge spasm also plays an important role in ST development. Therefore, we should monitor new-onset rest angina after stent implantation and carefully assess DES-induced coronary vasospasm.

## Background

Although drug-eluting stents (DES) reduce restenosis after percutaneous coronary intervention (PCI) more effectively than bare-metal stents (BMS), there are a variety of DES-related complications, such as coronary artery vasospasm. Some reports have suggested that endothelial dysfunction and enhanced vascular smooth muscle contractility with the involvement of the Rho-kinase pathway play an important role in the pathogenesis of DES-induced coronary vasospasm [1, 2]. Coronary vasospasm increases the risk of life-threatening cardiovascular events. An inability to administer adjunctive medical treatment for DES-induced coronary vasospasm after stent implantation promotes the coagulation system and thrombus formation, which may result in acute myocardial infarction [3, 4]. Here, we present a case in which DES-induced severe coronary vasospasm was clinically suspected as a cause of subacute stent thrombosis (ST).

## Case presentation

**Fig. 1:**
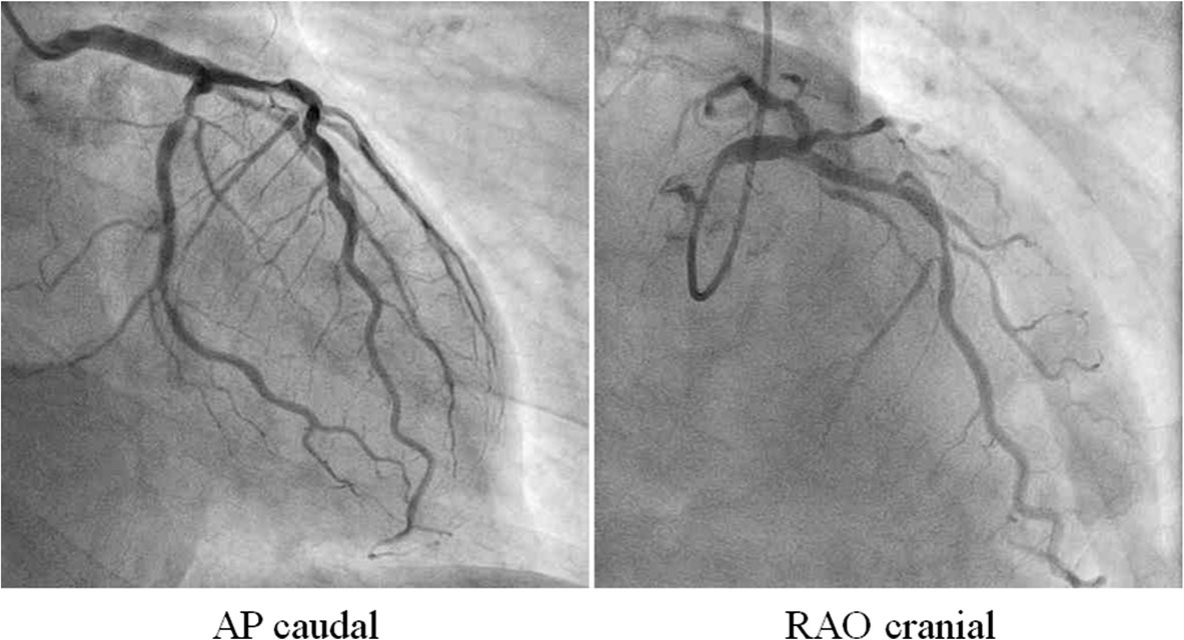
Pre-intervention coronary angiography. The left coronary artery showed intermediate stenosis in the proximal left ascending artery and left circumflex artery. Fractional flow reserve was 0.77 and 0.79 in the left ascending artery and left circumflex artery, respectively. *AP* anterior-posterior, *RAO* right anterior oblique

**Fig. 2:**
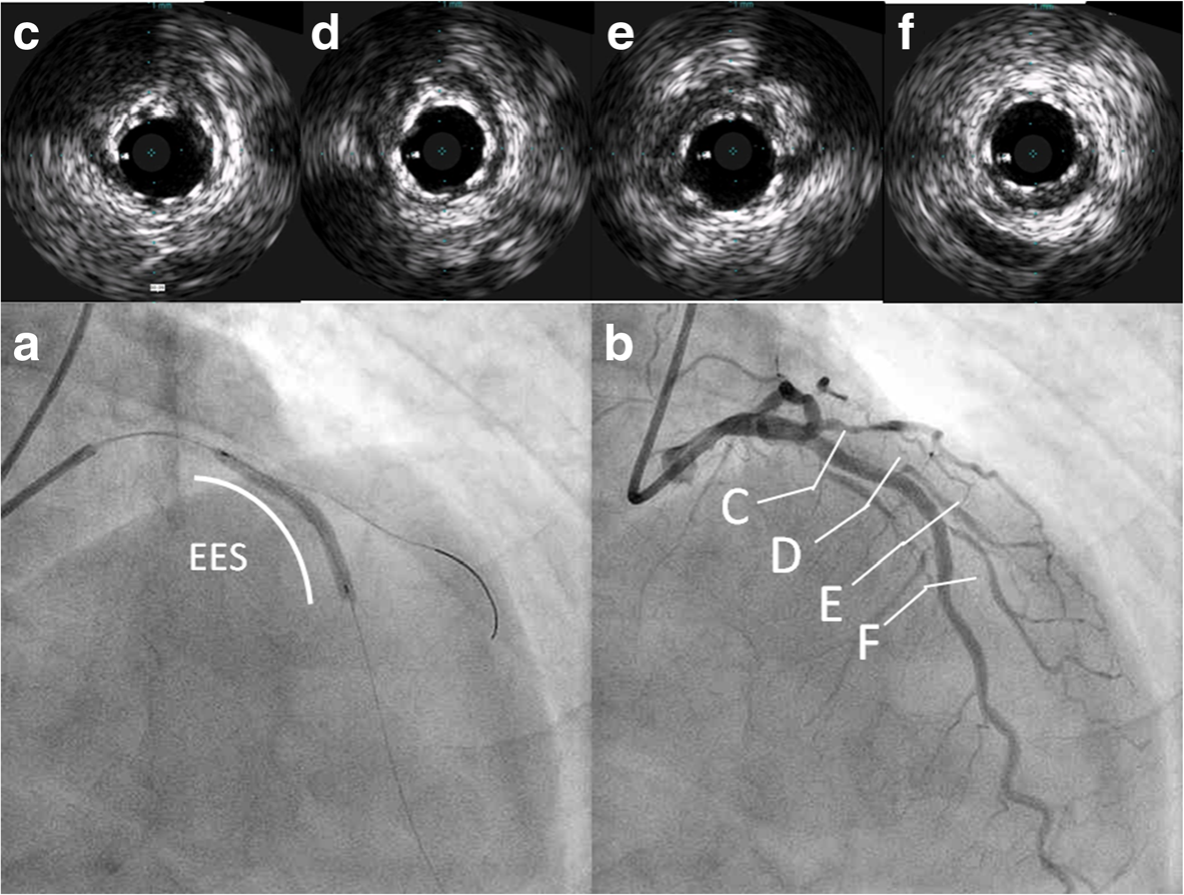
Elective percutaneous coronary intervention to the left ascending artery (**a** and **b**) and intravascular ultrasound findings (**c**-**f**). **a** Implantation of an everolimus-eluting stent (3.25 mm in diameter, 33 mm in length). **b** Final angiogram after post-dilatation, indicating good blood flow. **c**-**f** Intravascular ultrasound findings of the implanted everolimus-eluting stent (**c** proximal part of the stent, **d** part of first diagonal branch, **e** part of second diagonal branch, **f** distal part of the stent) indicating that the stent was well-expanded and without incomplete apposition

**Fig. 3:**
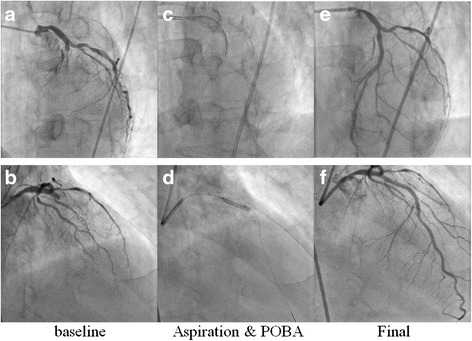
Emergent coronary angiography and percutaneous coronary intervention for subacute stent thrombosis. **a** and **b** Total occlusion of the proximal part of the stent with thrombus. **c** and **d** Aspiration thrombectomy and balloon angioplasty (high-pressure balloon, 3.25/15 mm) were performed. **e** and **f** Final coronary angiogram, indicating TIMI grade 3 flow. *POBA* plain old balloon angioplasty

**Fig. 4:**
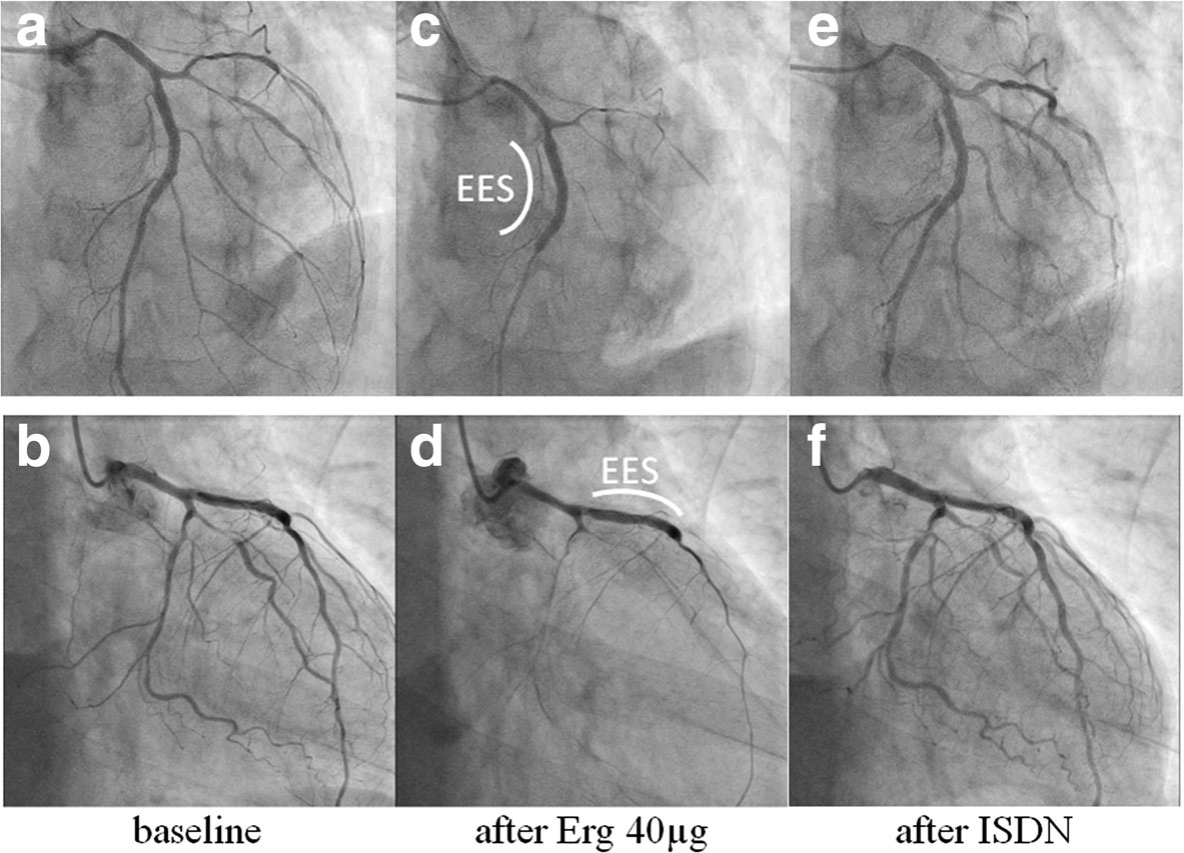
The spasm provocation test. **a** and **b** Baseline coronary angiography. **c** and **d** Intracoronary infusion of ergonovine induced a severe spasm in the left coronary artery, except for the stented lesion; the left circumflex artery was occluded. **e** and **f** Intracoronary infusion of isosorbide dinitrate completely resolved the coronary vasospasm. *EES* everolimus-eluting stent, *Erg* ergonovine, *ISDN* isosorbide dinitrate

A 67-year-old man presented with effort angina for one month. His coronary risk factors were current smoking and dyslipidemia, and his only regular medication was rosuvastatin. There was no significant past medical history. His baseline electrocardiogram (ECG) and echocardiography were normal, but coronary angiography (CAG) revealed intermediate stenosis in the left ascending artery (LAD) and left circumflex artery (LCx). We performed fractional flow reserve in the LAD and LCx; the results were 0.77 and 0.79, respectively (Fig. 1). We planned elective PCI to the LAD for effort angina. After starting dual anti-platelet therapy (DAPT) with 100 mg/day aspirin and 75 mg/day clopidogrel, successful angioplasty of the mid-LAD was performed. An everolimus-eluting stent (3.25 mm in diameter, 33 mm in length) was deployed with high pressure balloon (3.5/10 mm) post-dilatation (Fig. 2a and 2b). The final angiogram indicated good blood flow and the intravascular ultrasound indicated that the stent was well-expanded and there was no incomplete apposition (Fig. 2c-f). However, the patient reported new-onset chest discomfort at rest the next morning. The symptom resolved after a few minutes with no significant ECG changes and he was discharged. However, 5 days after the PCI, he was transported to our hospital by ambulance due to crushing chest pain while drinking. On admission, his ECG revealed ST-segment elevation in the precordial leads. In the emergency room, ECG monitoring detected ventricular fibrillation, and a counter-shock was successfully delivered. He then underwent emergent CAG, which revealed thrombotic total occlusion of the proximal LAD (Fig. 3a and 3b). We diagnosed subacute ST and performed PCI. Aspiration thrombectomy and balloon angioplasty with a 3.25/15 mm balloon were performed (Fig. 3c and 3d) and TIMI-3 flow was obtained (Fig. 3e and 3f). After these procedures, he was admitted to the intensive care unit, where we confirmed the resolution of his critical status. There were no major complications and his general condition was improving, but he subsequently reported frequent chest discomfort, especially during the night and in the early morning. Although no significant ECG changes were observed, we performed follow-up CAG and a spasm provocation test with ergonovine to evaluate the coronary vasomotor response 2 weeks after the initial PCI. The baseline angiography revealed no significant findings (Fig. 4a and 4b). However, intracoronary infusion of 40 μg of ergonovine into the left coronary artery provoked severe vasospasm from the left main trunk (LMT) to the LAD, except for the stented lesion, and total occlusion was detected in the LCx (Fig. 4c and 4d). He indicated the chest discomfort was the same as usual. An isosorbide dinitrate injection improved both the vasospasm and the discomfort (Fig. 4e and 4f). His condition was diagnosed as coronary vasospastic angina (VSA); treatment with calcium channel blockers and nicorandil resolved his symptoms. We evaluated his cytochrome P450 2C19 (CYP2C19) genotype and determined he had a CYP2C19 polymorphism and was therefore a poor metabolizer of clopidogrel. Thus, we replaced the 75 mg/day clopidogrel with 3.75 mg/day prasugrel. His post-discharge clinical course was uneventful.

## Conclusions

We experienced a case of DES-induced severe coronary vasospasm, which may have contributed to the development of subacute ST. Although single stenting of the LMT showed a comparable clinical outcome compared to two-stent strategy [5], in this case, the intravascular ultrasound on initial PCI showed no significant stenosis in the LMT and we deployed DES only in the LAD lesion. In addition, ECG changes when stent-edge spasm was provoked after injection of ergonovine showed axis deviation to the left. This might indicate ischemia of the ventricular septum induced by vasospasm occurring in the first major septal branch.

This case is clinically important because it describes the potential hazard of stent-edge spasm after stent implantation. The patient’s clinical course provides the following two important clinical suggestions. First, that DES implantation for effort angina can induce new-onset stent-edge spasm, and second, stent-edge spasm may have an important role in the development of subacute ST.

The first clinical suggestion is that DES implantation for effort angina can induce new-onset stent-edge spasm. A recent study showed that endothelial dysfunction and enhanced vascular smooth muscle contractility with the involvement of the Rho-kinase pathway play an important role in the pathogenesis of DES-induced coronary vasospasm [1, 2]. Coronary vasoconstrictive responses are enhanced at the edges of coronary segments implanted with DES compared with BMS. The incidence of provoked spasm is not different between patients with and without VSA before stenting [6]; therefore, it is important to be concerned about the occurrence of new stent-edge spasm in patients both with and without VSA. It is possible that repeated, broad, strong, and long stent-edge spasm results in new cardiac events, and death was reported in some patients with severe multivessel, non-intervention-related vascular spasm after DES implantation [7, 8]; adjunctive medical treatment is critical for these patients. Long-term administration of vasodilating drugs, such as calcium channel blockers and nitrates are useful for inhibiting coronary vasospasm. Among four major calcium channel blockers (benidipine, amlodipine, nifedipine, diltiazem) that effectively suppress VSA, benidipine has shown significantly more beneficial prognostic effects [9].

The second clinical suggestion is that stent-edge spasm may have an important role in the development of subacute ST. There are two possible reasons for this phenomenon. The first is that coronary vasospasm promotes coagulation and may lead to thrombus formation. Some studies focused on the relationship between coronary vasospasm and thrombus formation have observed accelerated platelet and coagulation activities induced by coronary artery spasms [3, 10]. Platelet aggregation in the coronary circulation causes coronary thrombus formation, which may result in acute myocardial infarction [4]. Several cases in which coronary vasospasm led to coronary thrombosis and myocardial infarction have also been reported [11, 12], supporting a potential mechanism underlying the phenomenon of the stent-edge spasm-related subacute ST in this patient. The second possible reason for the phenomenon of subacute ST secondary to stent-edge spasm is that this patient had few subacute ST risk factors except for the CYP2C19 polymorphism and the stent-edge spasm. CYP2C19 polymorphisms have been associated with weaker antiplatelet responses to clopidogrel, and nonresponsiveness to clopidogrel is an independent predictor of ST [13]. However, CYP2C19 polymorphisms are frequent, especially in Japanese individuals, and the incidence is estimated about 20–25% of Japanese population [14]. Therefore, multiple overlapping risk factors are required for the development of ST, even in patients that are poor metabolizers of clopidogrel. Several studies have evaluated the potential predictors of acute and subacute ST. Multiple risk factors related to devices, patients, lesions, and procedure have been suggested in the development of ST [15, 16]. Although this patient had a CYP2C19 polymorphism, he had no other significant risk factors for the development of subacute ST. Device-related factors (e.g., stent material and design) were not a problem. Regarding patient and lesion-related factors, this patient did not have acute coronary syndrome, old age, diabetes, or low left ventricular ejection fraction and the target lesion was non-complex with simple characteristics. Concerning procedure-related factors, the deployed stent had no morphometric (underexpansion or asymmetry) or morphologic (dissection, incomplete apposition, or tissue protrusion) abnormalities. Therefore, another mechanism beyond the above risk factors must have played a role in ST development in this case. It is possible that the patient’s platelet function was not adequately reduced despite the standard post-stent DAPT administration (100 mg/day aspirin and 75 mg/day clopidogrel) before PCI. In addition, the stent-edge spasm likely promoted the coagulation system and thrombus formation to increase the risk of ST.

Unfortunately, we lacked a baseline spasm provocation test before DES implantation and it is unknown whether coronary vasospasm was already present or not. However, the result of the fractional flow reserve evaluation showed myocardial ischemia and the patient’s symptoms also obviously changed from typical exertional chest pain to chest discomfort at rest after stenting. The clinical course indicated that his coronary vasomotor response was not clinically problematic before stenting, even if he already had coronary vasospasm. Therefore, we believe that coronary vasospasm became apparent after DES implantation and that stent-edge spasm should have been treated with antivasoconstrictive drugs to control and prevent the occurrence of subacute ST.

This case is clinically important because it describes the potential hazard of DES-induced coronary vasospasm, which may have caused subacute ST. Although various risk factors overlap to contribute to the development of ST, we believe stent-edge spasm also has an important role in ST development. Therefore, we should monitor new-onset rest angina after stent implantation and carefully assess DES-induced coronary vasospasm.

## Abbreviations

BMS: Bare-metal stents
CAG: Coronary angiography
CYP2C19: Cytochrome P450 2C19
DAPT: Dual anti-platelet therapy
DES: Drug-eluting stents
ECG: Electrocardiogram
LAD: Left ascending artery
LCx: Left circumflex artery
LMT: Left main trunk
PCI: Percutaneous coronary intervention
ST: Stent thrombosis
VSA: Coronary vasospastic angina
